# Development and use of machine learning algorithms in vaccine target selection

**DOI:** 10.1038/s41541-023-00795-8

**Published:** 2024-01-20

**Authors:** Barbara Bravi

**Affiliations:** Department of Mathematics, Imperial College London, London, SW7 2AZ UK

**Keywords:** Drug development, Vaccines

## Abstract

Computer-aided discovery of vaccine targets has become a cornerstone of rational vaccine design. In this article, I discuss how Machine Learning (ML) can inform and guide key computational steps in rational vaccine design concerned with the identification of B and T cell epitopes and correlates of protection. I provide examples of ML models, as well as types of data and predictions for which they are built. I argue that interpretable ML has the potential to improve the identification of immunogens also as a tool for scientific discovery, by helping elucidate the molecular processes underlying vaccine-induced immune responses. I outline the limitations and challenges in terms of data availability and method development that need to be addressed to bridge the gap between advances in ML predictions and their translational application to vaccine design.

## Introduction

Vaccine design is rapidly progressing from empirical to more systematic, rational strategies that benefit from computational predictions to assist the identification of pathogen regions targeted by the immune system (epitopes)^1^. Examples are reverse vaccinology approaches for the design of protein subunit vaccines^2^, which start from the genetic sequence of the pathogen and screen the possible antigens by their potential immunogenic and protective efficacy to select a few main targets. An accurate selection of targets is essential to imparting specific yet sufficiently immunogenic stimuli, while potentially avoiding antigens that do not elicit protective immunity. Since identifying epitope regions experimentally is resource and time-consuming, predictions in silico play the fundamental role of narrowing down the number of candidate targets to carry forward to in vitro and in vivo testing. As such, they will be key to rapid and cost-effective manufacturing of next-generation viral vectored or nucleic acid-based vaccines, first commercially developed during the recent Sars-Cov-2 pandemic^3^.

**Fig. 1:**
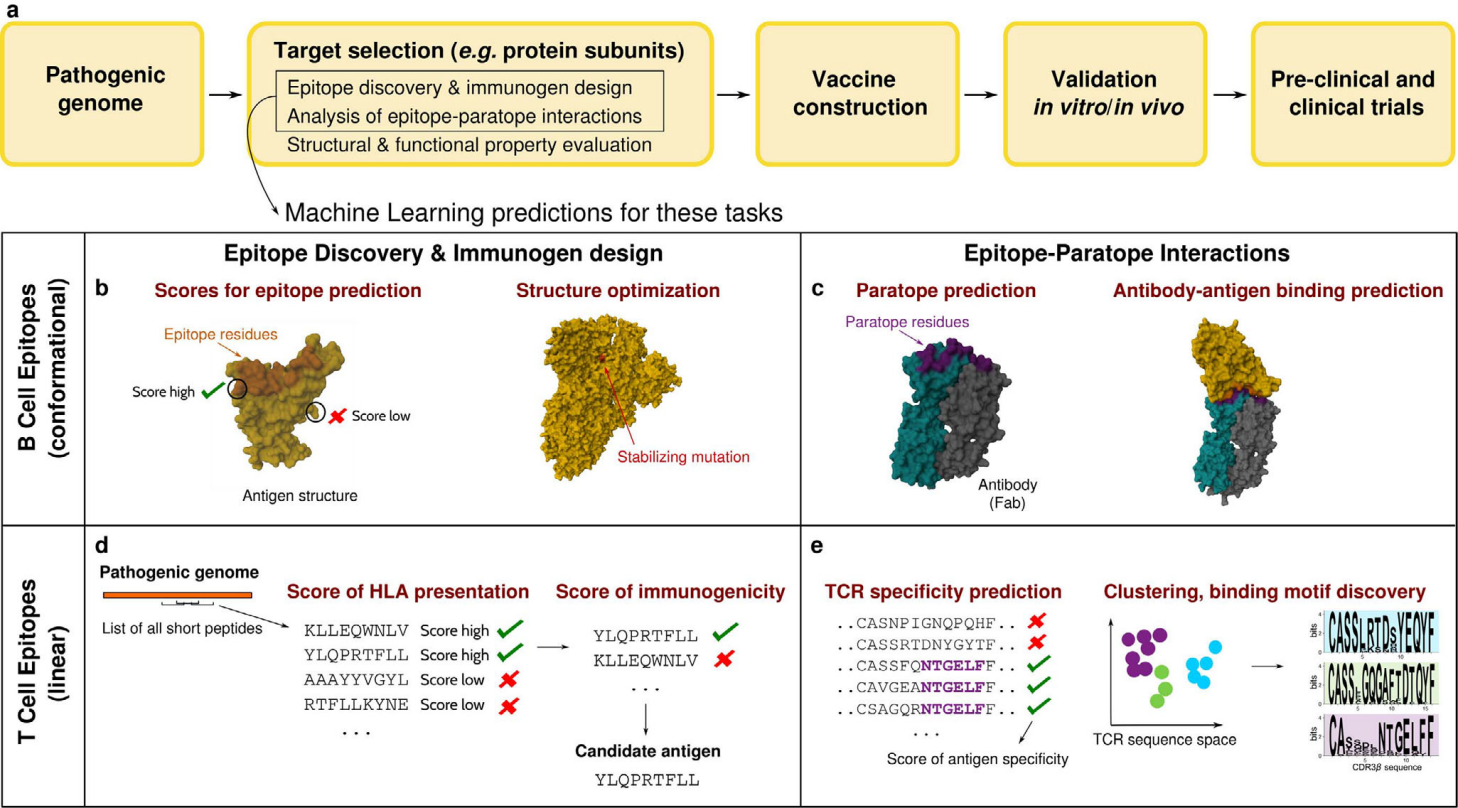
ML in vaccine target selection. Schematic of the rational vaccine design process (**a**) and machine learning applications to key tasks in vaccine target selection: B and T cell epitope discovery and immunogen design (**b**, **d**); characterization of correlates of protection through quantitative modeling of epitope-paratope interactions (**c**, **e**). Structures' images obtained with Mol*^199^.

Computational screening of putative targets can be performed via several bioinformatic tools (see for example refs. ^4,5^), made available on the Immune Epitope Data Base (IEDB)^6^ and other web servers^7–9^. The methods primarily gaining momentum and prominence among these tools are the ones from Machine Learning (ML), the ensemble of algorithms and model architectures to learn from data in such a way as to better analyze them and make new predictions (see Box 1 for the basic ML terminology). Several ML-based reverse vaccinology pipelines have been developed^7–15^, with promising applications to the prediction of bacterial protective antigens^7,10–12^ and Sars-Cov-2 antigens^8,13–15^. ML can assist several stages of vaccine design^16^, but its application is particularly key to a fast and accurate target selection during the initial phase (Fig. 1a). Here ML algorithms serve for the identification and optimization of B and T cell epitopes, and can inform the study of correlates of protection by helping assess quality and specificity of vaccine-induced cellular and humoral responses. Important questions in this regard concern which antibodies and T Cell Receptors (TCRs) bind to epitopes and trigger specific and high-magnitude responses, but also which of them can confer cross-variant immunity, a crucial question to formulate broadly protective vaccines for viruses undergoing fast antigenic drift like coronaviruses^17^. ML algorithms for epitope discovery, immunogen design, and prediction of epitope-paratope interactions have witnessed massive progress in recent years, spurred by fast-growing data availability and the latest developments in ML for protein modeling, standing out as illustrative examples of the potential advantages of ML in rational vaccine design.

Despite the success of conventional vaccination strategies, the molecular and cellular processes contributing to the efficacy and long-term protection of several vaccines are still not fully understood. In this regard, ML is emerging also as a tool for scientific discovery that, while delivering useful predictions for rational vaccine design, provides new methods for investigation in systems immunology and proteomics and can thus improve our understanding of immune responses across individuals.

In this article, I describe the current trends in ML methods for the discovery of B and T cell epitopes and for characterizing the response by the adaptive immune system to those epitopes at the molecular level. While comprehensive reviews of such methods are provided elsewhere^18–24^, my aim here is to describe the basic ideas, model architectures, and types of data involved in recent developments of ML in this context, as well as to discuss the prediction tasks and the biological insights made possible by them. I conclude with a brief overview of other ML predictions that are relevant to vaccine design (in vaccine construction and preclinical validation of selected vaccine targets), and with an outlook on current challenges and important directions for future work.

Box 1 ML basic terminology *Training set*: data used for training the model, i.e., to learn its parameters from the data. This learning procedure typically consists of finding the parameters that optimize (e.g., minimize) an ‘objective’ function (e.g., the error of classification or regression), and can be realized through a variety of algorithms (e.g., gradient descent algorithms).

*Test set*: data unseen during training used to evaluate the model’s performance.

*Supervised learning*: type of learning aimed at modeling an input-output mapping, where given output values for each input (labels) are used during training. Example tasks: regression and classification.

*Regression*: supervised learning task consisting in modeling the relationship between a (real-valued) outcome variable and some inputs, used to make predictions on the outcome variable.

*Classification*: supervised learning task consisting in the assignment of input data to their class (e.g., the class of positives or negatives in a binary classification task). Often classification methods predict probabilistic scores of class assignment, and classification performance in this case is typically measured by the AUROC.

*AUROC*: Area Under the Receiver Operating Characteristic curve. In a binary classification task, the receiver operating characteristic curve plots the fraction of false positives vs the fraction of true positives varying the threshold in the predicted scores used to discriminate positives from negatives. The area under this curve (AUROC) is often taken as a summary metric of classification performance, as it gives the probability by which positive hits are well predicted by assigning to them higher scores than to negatives (AUROC=1 stands for perfect prediction, AUROC=0.5 stands for the random expectation).

*Unsupervised learning*: type of learning where no given labels are used during training, aimed at analyzing structure and properties of the data. Example tasks: clustering and dimensionality reduction.

*Clustering*: unsupervised learning task of sorting data points into different groups based on intra-group similarities.

*Dimensionality reduction*: unsupervised learning task where correlations and patterns in high-dimensional data are used to find a representation of data points in a low-dimensional space (i.e., specified by a small number of coordinates).

*Semi-supervised learning*: type of learning where labels are given only for a portion of the training set.

*Overfitting*: outcome of training whereby the model reproduces well the features of the training set but lacks generalization power, i.e., the power to predict well unseen data in the test set.

## Machine learning in immunology

The development of ML methods for immunology has been fueled by the production of large-scale immune repertoire and immunopeptidomic datasets, and their systematic collection and annotation in specialized databases^6,25,26^. These data provide information on the central proteins involved in immune responses (antibodies, TCRs, antigens), represented in terms of their sequence and/or structure (see Box 2 for a description of protein representations that are relevant to modeling immune protein data).

Computational techniques of ML applied to large immunological datasets can detect statistical patterns reflecting structural and functional properties, and can leverage them to learn models of the mapping between a given input (like a protein sequence) to the structural or functional property (like the protein’s binding specificity). Learning (or training) a model consists of iteratively adjusting its parameter values on the available training data (Box 1) in such a way as to achieve a certain prediction task, a procedure which is typically expressed mathematically as the optimization of an appropriately defined objective function. Once trained, the model can be evaluated on new data, enabling novel predictions and insights fully in silico.

Model training can be performed in a supervised way (Box 1), like for classification tasks (e.g., classifying epitope vs non-epitope protein sites) and regression tasks (e.g., predicting the antigen-antibody binding affinity); or in an unsupervised way, like for clustering tasks (e.g., grouping TCR sequences with similar binding motifs). Hence ML is appealing for its predictive and exploratory power, which helps build accurate prediction models and facilitates the inspection and discovery of biologically meaningful features.

The ML predictions related to epitope discovery, immunogen design, and prediction of epitope-paratope interactions are typically formulated in terms of ‘scores’, quantifying for example the probability that a given residue belongs to a conformational epitope (Fig. 1b) or the probability of peptide presentation and immunogenicity (Fig. 1d). Assigning these scores enables a fast ranking of candidate targets and the subsequent prioritization of a few. It also accelerates additional in silico studies relying on more computationally intensive methods, like molecular dynamics.

Several ML architectures have been applied in this context (Box 3), which differ by mode of learning supported (e.g., supervised vs unsupervised), type of prediction (e.g., regression vs clustering), expressive power (the ability to capture non-linear relationships and correlations in the data) and interpretability of the predictions obtained. In general, the choice of a certain ML architecture (e.g., a transformer vs a convolutional neural network) and of its specific structure (e.g., the number of its internal layers, setting the number of parameters to learn) is motivated by the specific prediction task to achieve and by the type and quantity of data available for training (for example, more parameters increase the model’s expressive power but may lead to overfitting). I will provide an illustration of these model selection aspects while introducing ML approaches to predicting B and T cell epitopes and epitope-paratope interactions.

Box 2 Protein representations The way in which we represent input data for modeling purposes has a crucial impact on the information we are able to extract from them. Protein modeling approaches are mainly divided into sequence-based and structure-based, depending on whether the protein data are represented by the set of the protein’s constitutive amino acids (sequence), each of them being denoted by a letter, or by the spatial coordinates of the amino acids’ atomic constituents (protein structure). The sequence representation is typically useful for the retrieval and analysis of sequence motifs, given by recurring groups of amino acids bearing functional significance (e.g., epitope-paratope binding sites, see Fig. 1e). The structure representation provides access to multiple potential levels of description, e.g., the global topological organization of the protein fold, structural motifs (like *α*-helices and *β*-sheets), protein surface characteristics, and residue-residue connectivity. Structure-based representations concentrating on residue-residue connectivity are often informative enough for functional characterization of protein sites while being more parsimonious (hence computationally less demanding), because connectivity encodes information related to molecular shape and flexibility, local residue motions upon ligand binding and allostery. Representations of this type are graph-based representations, which model atoms or residues as nodes of a graph, while edges between nodes recapitulate closeness in space and chemical bonds. As such, they are also well-suited to build ML models that can capture local symmetries and generate predictions that are invariant under geometrical transformations like rotations^51^.

**Fig. 2:**
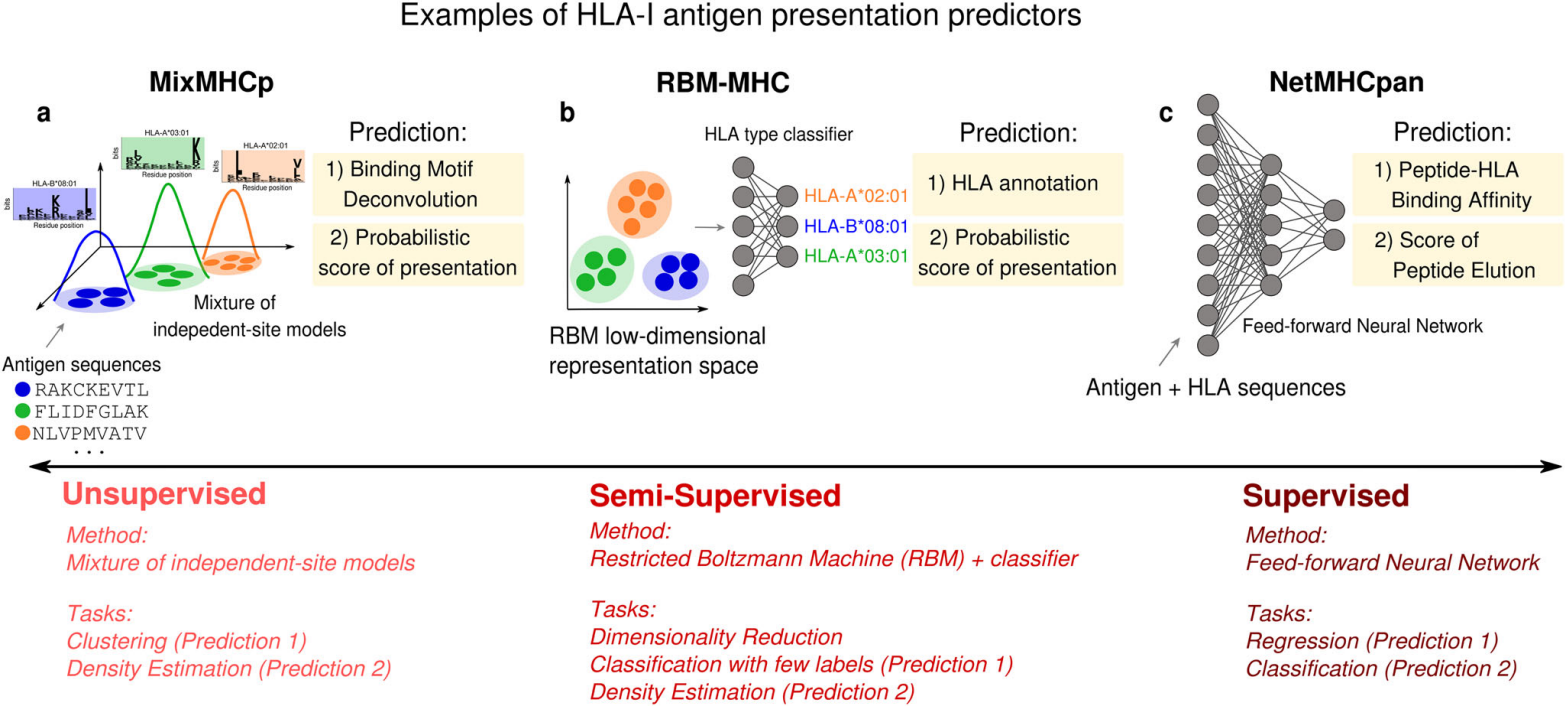
Antigen presentation prediction. Examples of predictors of HLA class I antigen presentation that are based on different types of ML methods: **a** MixMHCp^79,81^ is an unsupervised method using a mixture of probabilistic independent-site models to perform clustering of peptides and binding motif deconvolution; **b** RBM-MHC^89^ is a semi-supervised method relying on a dimensionality reduction step (performed through an RBM model) to leverage small amounts of antigens labeled by their HLA specificity to train an HLA-type classifier; **c** NetMHCpan^87,88^ is based on a supervised feed-forward neural network trained on antigen and HLA sequences to predict peptide binding affinity (from affinity data) and a score of peptide elution (from mass spectrometry eluted data).

The choice of a representation depends on reasons of data availability, computational expediency, and is informed by domain knowledge, which can suggest the data characteristics (‘features’) or the level of approximation most adequate to a given prediction task. The main idea behind feature-based ML is to select and design sets of features to use as data representations that are fed into a ML method as inputs. Features to describe protein regions of interest typically summarize their biochemical (for example, hydrophobicity, polarity) and geometrical (for example, surface area, accessibility) properties. A heuristic, hence approximate, choice of features can be however labor-intensive and has inevitably limited predictive power. A novel approach enabled by ML is the one of *learning* data representations that are discriminative for prediction. ‘Representation’ in this context is meant as the outcome of learnable transformations applied by the ML model to each data point, ahead of computing the final output. It consists of a vector of numerical values specifying the data coordinates in the model’s representation space (it is usually referred to also as ‘vector embedding’). Examples are: the low-dimensional representations used by RBMs (Box 3) for dimensionality reduction, which has been leveraged to predict antigen-HLA specificity^89^ (Fig. 2b); the high-dimensional vector embeddings of language tokens learnt by language models (Box 3) to capture fine-grained contextual information, which has been leveraged to predict B cell epitope residues^36^. The key advantage of mapping data onto a representation space is that vicinity in this space reflects similarities between data points, for example, for proteins, similarity in sequence composition or in the structural arrangement. As such, the organization of protein data in this space is informative about shared structural and functional properties and phylogenetic relationships, facilitating subsequent prediction tasks as well as data exploration and interpretation^152^.

There is a subfield of ML, ‘representation learning’^200^, concerned precisely with the design of ML strategies to learn informative, useful, and meaningful data representations, hosting active research on representation learning for proteins^201^.

Box 3 ML architectures *Feed-forward neural network*: a neural network is a ML model consisting of: an input layer (a set of units representing the single components of the input data, e.g., for protein sequences, the residues’ identities or physico-chemical properties); an output layer (a layer where each of its units stands for a model’s prediction); usually an additional stack of intermediate layers of units (called hidden layers). A feed-forward neural network is one of the most common neural networks, where the units in each layer are connected only to the units of the following layer through a set of parameters (weights) learnable during the model’s training, in such a way that the information flows only in the forward direction (from the input to the output through the hidden layers), see for example Fig. 2c. Each hidden layer implements a transformation of the output from the previous layer through a typically non-linear learnable function; the non-linearity of such transformations is key to their ability to learn complex input-output functions.

*Restricted Boltzmann Machine (RBM)*: generative ML model whose architecture consists of an input layer connected to only one hidden layer, see for example Fig. 4d. This architecture parametrizes a probability distribution over the input data and the hidden units (from which the probability of the data can be retrieved by marginalization over the hidden units). The hidden layer is useful for increasing the model’s expressive power and for dimensionality reduction, see for example Fig. 2b.

*Deep learning*: ML methods relying on neural network architectures with multiple hidden layers.

*Convolutional Neural Networks (CNNs)*: neural networks containing convolutional layers, firstly developed for applications in computer vision. A convolutional layer implements a transformation called convolution between a region of the input and a small matrix of learnable weights (filter), which is progressively swept across the input. The use of the same small filter enables the detection of localized features and the equivariance of predictions (i.e., when input features are translated the output of the convolutional layer is translated consistently), which ensures that feature detection is robust to its exact position.

*Decision tree*: ML algorithm generating a tree-like structure through a series of decisions based on the input features, which serve to obtain the final classification or regression prediction, see for example Fig. 4a. One of the most popular applications is within methods that train ensembles of decision trees and combine their predictions to gain robustness and generalization power, like random forests.

*Language models*: ML architectures developed to model relationships in language data, like sentences, used for language processing tasks such as machine translation, keyword recognition and text generation. Language models are currently widely adopted in protein modeling, where protein sequences are treated in analogy to sequences of text symbols. One of the most powerful language model architectures is the transformer, a neural network which processes sequences of symbols by alternating attention-mechanism layers and non-linear transformations. The transformer is increasingly preferred to other established language models, like Recurrent Neural Networks (RNNs) and Long Short-Term Memory (LSTM) networks, due to its ability to effectively capture long-range dependencies between symbols and hence to produce fully contextual representations.

*Attention mechanism*: after its introduction in the transformer architecture, it has become a key building block of language models and other deep learning architectures. The attention mechanism assigns to each input component (like a text symbol) a score quantifying its relevance to the context of the other input components, based on the statistical dependencies detected. The set of these scores for the different input components forms an attention map (see for example Fig. 4c).

*Generative models*: models that perform density estimation, i.e., they reconstruct the probability distribution from which the data have been generated, supporting the design of synthetic data by sampling from the learnt distribution. For sequence data, generative models range from probability distributions obtained simply from the frequencies of symbols at each position (independent-site models, see for example Fig. 2a), to probability distributions specified by shallow ML architectures like RBMs or by deep generative language models based on transformers, RNNs and LSTMs. Other generative ML architectures increasingly employed in protein modeling are variational autoencoders^202^, generative adversarial networks^203^, and diffusion models^204^.

## B and T cell epitope discovery

### Prediction of B cell epitopes

Broadly speaking, the ML methods used for linear and conformational B cell epitope prediction are trained in a supervised way to discriminate epitope sites from generic ones that are typically not targeted by B cells, outputting an epitope likelihood score for each site^27–36^ (Fig. 1b). B cell epitopes are predominantly conformational, hence their prediction is better supported by methods trained on protein structures (Box 2), which can exploit information on the antigen surface topology in addition to the biochemical composition provided by the sequence.

In general, ML for B cell epitope discovery builds upon feature-based ML, which performs a key preliminary step of feature selection and engineering (Box 2). The intuition behind this is that only a few sequence and structure properties contribute to determine whether a residue is an antibody binding site. Indeed, residues’ physico-chemical properties have been suggested to favor the maturation of high-affinity antibodies, and have been used for epitope identification also before the advent of ML^37,38^, along with conformational properties such as flexibility^39^, residue protrusion^40,41^, and surface accessibility^42^. Feature selection enables as well to reduce the dimensionality of the input data (otherwise specified by thousands of atomic coordinates), with gains in computational efficiency. In conformational epitope discovery, these features typically consist of physico-chemical attributes (e.g., hydrophobicity and electrostatic potential^28,33^), high-level geometric properties (e.g., type of secondary structure^28,33^, solvent accessibility and average curvature of the molecular surface^28^), evolutionary information (e.g., conservation^28,33^), and specific combinations of amino acids in pairs or triplets^29^.

Graph-based representations (Box 2) of epitope regions have also been used in this context along with residue physico-chemical properties^29,33^. ML approaches based on graph-theoretical descriptors have been successful at protein design^43,44^, identifying interaction sites^45–49^, and predicting the effect of mutations^50^: all these works provide additional examples of feature selection and learning strategies that could be adapted to the epitope identification problem as this field progresses and new data become available. The motivation for developing graph-based approaches to epitope identification is that epitope regions exhibit distinctive signatures (in terms of residue packing as well as type and topological arrangement of bonds) that can be conveniently summarized by a graph representation^29,33^. An advantage of graph-based ML is that it can leverage efficient algorithms from the well-established field of graph theory^51^. The challenge however remains of determining the appropriate scale for constructing the graph (e.g., atom vs residue level), and the information to embed in the definition of graph links (e.g., whether weighting them by geometrical characteristics of the modeled protein region^29^). The design of graph-based descriptors, and more generally feature engineering, depend on our understanding of the most relevant features, which can render the predictions prone to bias due to over-reliance on certain properties commonly associated to the functional behavior of interest (e.g., a protrusive instead of planar surface for epitopes^29^). Even if correlations of epitope propensity to chemical and geometrical features have been established, an open question is how they should be combined when used as inputs of ML algorithms to achieve an accurate epitope prediction. At present, there are no general guiding principles to address this problem, which is mainly dealt with by careful and potentially very time-consuming work of systematic feature elimination and search over feature combinations.

A new approach that has led to substantial gains in performance at B cell epitope identification is the one of learning protein representations tailored to the B cell epitope prediction task (Box 2). Ref. ^35^ has pioneered this approach, using deep learning to build representations of spatio-chemical arrangements of residues’ neighborhoods that are informative about protein-protein binding and epitope recognition (see also section Interpretable machine learning approaches). Another approach recently proposed^36^ is to appeal to residue-specific representations extracted by protein language models (Box 2), learnt in such a way as to embed contextual information (the rest of the sequence and the backbone structure^52^), and use them as information-rich inputs to train a ML epitope predictor. The key idea behind this approach is that the unsupervised learning of language models from massive protein datasets discovers inter-residue dependencies that are not captured by handcrafted features, and that can be leveraged for the downstream task of B cell epitope prediction, reaching a performance AUROC ~ 0.8 ^36^ (Box 1).

In general, efforts of structural characterization of the targeted protein, already pursued through comparative protein structure modeling^53^ and protein-protein docking^54–57^, can serve to optimize the antigen-antibody interaction surface^1,58^ (Fig. 1b), and to identify amino acid substitutions conferring enhanced conformational stability and expression (for example the 2 proline mutations at positions 986 and 987 for the Sars-Cov-2 spike protein, included in several COVID-19 vaccines^59^). ML has the potential to assist this task by identifying residues most involved in conformational variation^60^, whose mutations can be further studied via molecular dynamics, or by predicting free energy changes upon residue mutations^61–63^. While the performance of the later approaches seems stagnating^63^, recent progress in deep learning-based protein design holds promise to be useful at proposing expression and stability-enhancing mutations^64^.

On the other hand, ML predictors of B cell epitopes that are sequence-based^27,30–32^ (Box 2) are more convenient than structure-based ones, due to their higher computational speed. Despite having typically lower performance compared to structure-based ones (AUROC slightly above 0.75 for the example of state-of-the-art method of ref. ^31^), they enjoy a wider and more flexible scope of application given the large number of protein sequences available compared to structures. They are better-suited for linear B cell epitopes, but they are potentially useful also for conformational ones by capturing, thanks to the context-aware representations from protein language models (Box 2–3), functional dependencies between amino acids far apart along the sequence but proximal in the 3D structure^31^.

ML methods like AlphaFold^65–67^, trRosetta^68^, and RoseTTAFold^69^ can bridge this scale gap between sequence and structure data availability by enabling predictions of protein structure from sequence alone with unprecedented accuracy. Predictors of protein structure have huge potential still to be fully explored for the design of immunogens guided by structural insights^59,70^, as well as for antibody and TCR engineering. Antibody-specific predictors have been proposed^71–74^ based on deep learning architectures similar to AlphaFold, TrRosetta and RoseTTAFold. A specialized version of AlphaFold has been developed to study the structural interactions of the molecular complexes antigen-TCR^75^. In addition, ML-predicted structures are used for the complementary task of data augmentation, i.e., to enlarge the available training and test sets^36,74,76^. However, paratope, epitope, and in general functional site identification remains challenging even with the availability of these methods; for instance, the prediction of epitope-paratope binding sites by Alphafold-Multimer^67^ (the AlphaFold method tailored to protein complexes) was found to be inaccurate^35,67^.

### Prediction of antigen presentation

Protein targets of T cells are presented on the cell surface as short linear epitopes by the Human Leukocyte Antigen (HLA) complexes, with the epitopes of killer T cells presented in the context of HLA class I (HLA-I) molecules and the ones of helper T cells presented by HLA class II (HLA-II). Antigen presentation is the most selective step determining what pathogenic protein regions are likely to be targeted by T cells, hence its computational prediction is key to filtering effectively candidate targets for vaccine design (Fig. 1d). For example, the proteome of SARS-CoV-2 harbors ~ 10^4^ potential 9-mer HLA-I antigens. Bioinformatic analyses typically seek for ~ 1% of these peptides as predicted presented antigens per HLA allele^77^, corresponding to general estimates of the viral peptidome fraction that binds to HLAs^78^.

Figure 2 illustrates how the different ML concepts and methods in Box 1 and Box 3 have been adapted to the prediction of HLA-I antigen presentation (see also refs. ^18,19^ for comprehensive reviews). Existing ML predictors range from unsupervised clustering methods to perform binding motif deconvolution from unannotated eluted ligand data, like MixMHCp and MixMHCpred^79–81^ (Fig. 2a), to feed-forward neural networks trained in a supervised way to predict peptide presentation from known peptide-HLA pairs, like MHCflurry^82,83^ and the NetMHC and NetMHCpan suites^84–88^ (Fig. 2c). An alternative approach is RBM-MHC^89^, which addresses the problem of assigning antigens to their respective HLA-I molecule in newly produced or custom immunopeptidomic samples by resorting to a semi-supervised strategy (Box 1). The ML architecture here (a Restricted Boltzmann Machine, RBM, see Box 3) internally transforms sequence data onto a lower-dimensional representation, which facilitates the task of annotating antigens by their HLA-I type, since in this representation space antigens cluster by their HLA-binding motifs. Such a cluster structure enables to build an accurate predictor of HLA specificity using only a small amount of HLA-annotated antigen data from public databases (Fig. 2b). In addition, MixMHCp and RBM-MHC (Fig. 2a, b) learn generative models (Box 3), i.e., they estimate the probability distribution describing the immunopeptidomic data, assuming a different parametric form for such a distribution (respectively, a mixture of probabilistic independent-site models and an RBM). This peptide sequence probability can be used as a probabilistic score of presentation to distinguish presentable from generic non presentable sequences.

The data used to train these methods are HLA-antigen binding assays and eluted peptidomic data obtained via mass spectrometry, to a large extent publicly available in the database IEDB^6^. Recently there has been a shift towards an increasing use of eluted data from mass spectrometry^83,88,90–94^, which allow one to machine-learn information about all the steps of HLA-mediated processing and presentation^83,92,94^, and not only peptide binding affinity to the presenting HLA. For instance, the most recent versions of NetMHCpan have been tailored to integrate both data types to boost performance^87,88^ (Fig. 2c).

Currently, HLA polymorphism remains an unmet challenge for HLA-I presentation prediction. Most of the methods achieve near-perfect prediction for common HLA alleles, but perform poorly for rarer alleles. Improving the accuracy of predictors across all HLAs is key to ensuring high HLA coverage of vaccines across human populations. This problem has motivated the development of methods that use information on the HLA sequence to deliver HLA pan-specific predictions, like MHCflurry 2.0^83^ and NetMHCpan methods^87,88^ (Fig. 2c), and methods that can be easily re-trained by the user on newly available HLA-specific datasets^89^.

Predicting the presentation by HLA-II is much more challenging due to limited data availability and the diversity of allele-specific binding motifs. The data used for training the predictors are still limited to a few alleles (mainly from the genetic locus HLA-DR), and precisely the increased quantity of data on HLA-II presented peptides has been key to the latest improvements in prediction performance^91,93,95–97^, especially for less well-characterized alleles^97^. Binding-motif diversity is two-fold: first, alternative binding modes for the same HLA allele, including binding in the reverse peptide orientation, have been documented^96^; second, HLA-II presented peptides exhibit substantial variability in length (12-25 amino acids, compared to 8-14 for HLA-I), with multiple peptides of different length sharing a similar binding core at a variable starting position. To deal with this difficulty, state-of-the-art methods^91,93,95^ implement a dynamical search for the binding core within each peptide, either by scoring different sliding motifs along peptides^93,95^, or by appealing to the ability of Convolutional Neural Networks (CNNs, Box 3) to detect features regardless of their exact location^91^. Currently positive hits are distinguished from negative ones with AUROC in the range 0.8-0.85 at best, indicating that there is still room for improvement in performance.

### Prediction of antigen immunogenicity

Only a subset of HLA-presented antigens is immunogenic, so a few computational and ML methods have been proposed to predict which presented antigens tend to promote a T cell response and are likely to be immunodominant. Predictors of T cell epitope immunogenicity typically compare presented antigens that are immunogenic to non-immunogenic ones to estimate scores of immunogenicity, both for HLA-I^98–104^ and HLA-II peptides^101,105^. Such scores can be predicted based on the single-site amino acid enrichment in immunogenic vs non-immunogenic antigens^98,99^ or by supervised ML methods that are trained to discriminate them, using only sequence information^100,101,103–105^ or including also the peptide-HLA complex structure^102^. In these studies, the propensity to TCR binding has been correlated to physico-chemical properties of the peptide’s side chains facing out from HLA binding groove, such as hydrophobicity and aromaticity, and based on this observation some predictors select a priori peptide positions^98,99,103,104^ or amino acid properties^100^ deemed to be important to immunogenicity. The ML approach we have recently proposed^106^ models immunogenicity by learning the statistical differences in amino acid composition between immunogenic and presented-only antigens, avoiding the need for data validated as non-immunogenic, and recovering, instead of assuming a priori, the peptide positions and properties more frequently involved in TCR response.

The prediction of T cell epitope immunogenicity is of particular interest in pipelines of neoantigen discovery for the design of T cell-based anti-cancer vaccines^107–109^. Prediction methods here need to take into account immunogenicity-determining factors specific to immunity in cancer, such as low cross-reactivity with self-antigens and clonality of mutations. A recent large-scale validation of existing predictors used for neoantigen discovery has highlighted the need for substantial improvement in their performance^110^.

Indeed, in general, immunogenicity prediction methods have maximal AUROCs ~ 0.7^103,106^ (hence lower than for B cell epitope prediction), and in particular the performance becomes poor beyond a few immunodominant epitopes presented by common HLAs^21^. A main shortcoming is that the biological parameters determining immunogenicity, and hence to account for in a ML model, remain to be understood. For example, there is no consensus regarding whether high affinity and stability of binding to the HLA is correlated to high immunogenicity^111^, observed in some settings^112,113^ but not in others^114^. A paratope-agnostic identification of epitope sites, which side-steps the details of specific epitope-paratope interactions, has clear advantages in a translational setting of vaccine or therapy design but has also limited predictive power, because sequence and structure of the target protein are not the only determinants of a positive immune response. Modeling epitope-paratope interactions is hence crucial to improve epitope prediction, as well as to characterize more globally correlates of immune response upon vaccination (Fig. 1c, e).

## Epitope-paratope interactions

**Fig. 3:**
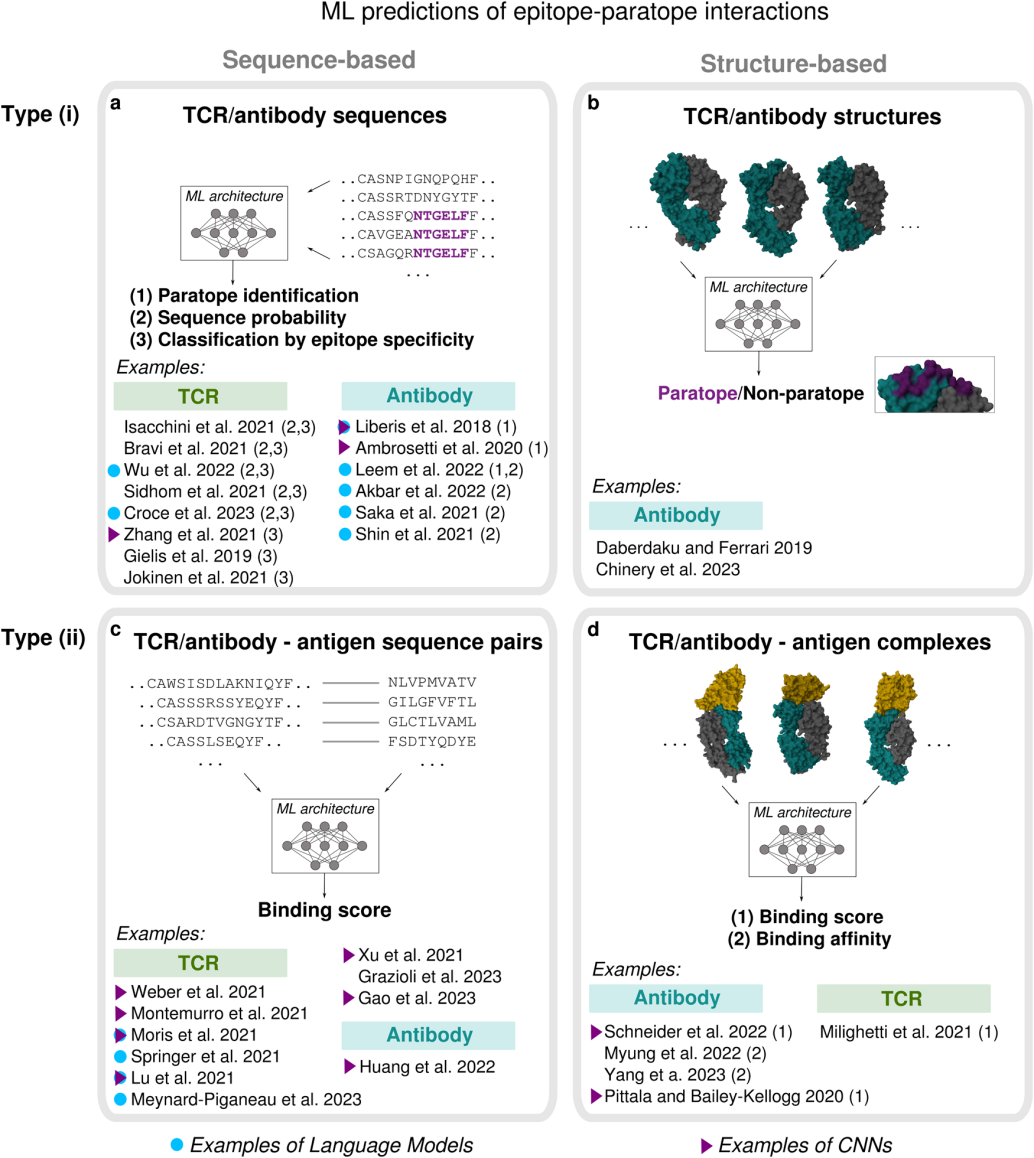
Epitope-paratope interaction prediction. Scheme of ML methods to predict epitope-paratope interactions for B and T cells, organized in terms of type of input: applicable to TCR/antibody sets only (**a**, **b**) vs TCR/antibody-antigen pairs (**c**, **d**); sequence (**a**, **c**) vs structure-based (**b**, **d**). Structures' images obtained with Mol*^199^.

Another area relevant to rational vaccine design is modeling through ML the specificity of epitope recognition both by TCRs (as reviewed in refs. ^22,115^), and antibodies (as reviewed in refs. ^23,24^). These ML models are trained on: (i) sets of TCR/antibody only (Fig. 3a, b); (ii) TCR/antibody-antigen binding pairs (Fig. 3c, d).

Examples of ML models of type (i) are the ones built for in silico paratope identification in antibodies, based on the assumption that the position of the paratope is largely antigen-independent. ML methods here are trained to classify an antibody residue as part or not of the paratope, estimating for each residue a probabilistic score of belonging to it^76,116–118^; their performance is currently quite high (AUROC above 0.9, see one of the latest comparisons in ref. ^76^). In silico paratope identification is relevant especially to antibody design, since it helps propose putatively binding-improving mutations^72^. Other main examples of ML models of type (i) are generative models (Box 3) learnt from TCR/antibody sequences (Fig. 3a), which estimate a sequence probability distribution^119–126^. Generative models are generally of great interest to the field of molecular design: sampling from the learnt distribution allows one to generate putatively functional synthetic data, for instance antibodies with optimized binding properties^124–126^. Several ML models of type (i) are trained on sets of TCR sequences binding to the same antigen, predicting scores to classify new TCRs as specific or unspecific to the corresponding antigen^119–122,127–130^ (Fig. 3a). Some of these approaches can also detect recurrent amino acid motifs in TCRs that are the statistical signature of antigen-binding specificity^120,122,128^, similarly to the clustering methods designed for binding motif discovery^131–133^ (Fig. 1e).

Models of type (ii) attempt to model the specific interactions involved in epitope-paratope binding. They can typically predict binding scores, that are able to discriminate epitope-paratope binding pairs from non-binders^134–143^. These predictions are useful to characterize antigen specificity of unseen TCRs^134–140,143–146^, to identify paratope and epitope sites^147^, and to accelerate further analyses through docking algorithms, e.g. by improving the selection of docking poses^142,148^. Such binding predictions can inform vaccine design, because they enable the screening in silico of putative antigen targets against large sets of TCRs and antibodies, thus helping characterize them in terms of elicited response, dominance and prevalence.

Similarly to conformational epitope discovery, structure-based methods for epitope-paratope interactions (Fig. 3b, d, Box 2) generally rely on a first step of feature selection, which extracts and embeds into feature variables their physico-chemical and geometrical properties^118,143,147–149^, including graph-based representations of the interface regions^76,147,148^; ML predictors of epitope-paratope binding are then trained on these features.

Epitope-paratope interactions are mediated by binding motifs that vary position and composition-wise across antibody-antigen pairs, as a consequence also of the variability in length of the Complementarity Determining Regions (CDRs). Identifying such motifs calls for prediction tools that are able to leverage information from residue neighborhoods and detect spatially localized features independently of their exact position. This type of prediction resembles the object recognition task in computer vision, where the state-of-the-art ML tools are CNNs (Box 3). CNNs have become a main trend in ML architectures for structure-based epitope-paratope binding^142,147^ along with neural networks designed to process graph-shaped inputs^76,148^. The richness of structural information enables the prediction of antigen specificity in TCRs with a performance comparable to sequence-based methods, despite the smaller training datasets^143^. It enables also to model the mapping between the antibody-antigen complex structure and its binding affinity^148,149^ (Fig. 3d), with a performance, estimated through the correlation coefficient between true and predicted affinity values, of up to 0.79^149^.

Sequence-based methods (Fig. 3a, c, Box 2) appeal as well to deep CNNs^116,117,128,134,135,137,138,140,141,144,145^, while in general spanning a variety of ML architectures, from decision trees and random forests^129,150,151^ to networks based on the attention mechanism^122,134,139,140^ (Box 3). Most recently, sequence-based methods have benefitted from the breakthroughs in ML for natural language processing, with several methods for epitope-paratope interactions and paratope prediction directly using language model ML architectures^116,121,123–126,130,136,137,146^ (Box 3). These architectures capture potentially long-range dependencies between residues along the sequence, resulting in representations of each protein site capable of incorporating the effect of the physico-chemical context^152,153^.

A recent public benchmark of sequence-based methods to predict TCR-epitope specificity has flagged up a few important trends^115^. Firstly, data set the performance to a larger extent than the particular model architecture. Indeed, the generalization power of different methods is consistent across antigens, with typical AUROCs in the range 0.7–0.9, and is correlated to the heterogeneity in sequence composition of TCRs binding to the same antigen. Secondly, predicting antigen specificity based on the TCR sequence similarity provides already a good baseline performance, in line with the observation of enriched sequence motifs in TCR sets with a given antigen specificity. Finally, the gain of deep learning over simpler models seems modest with the available data. Given that training deep learning models is data-demanding, tests on larger datasets are needed to clarify this point.

## Interpretable machine learning approaches

**Fig. 4:**
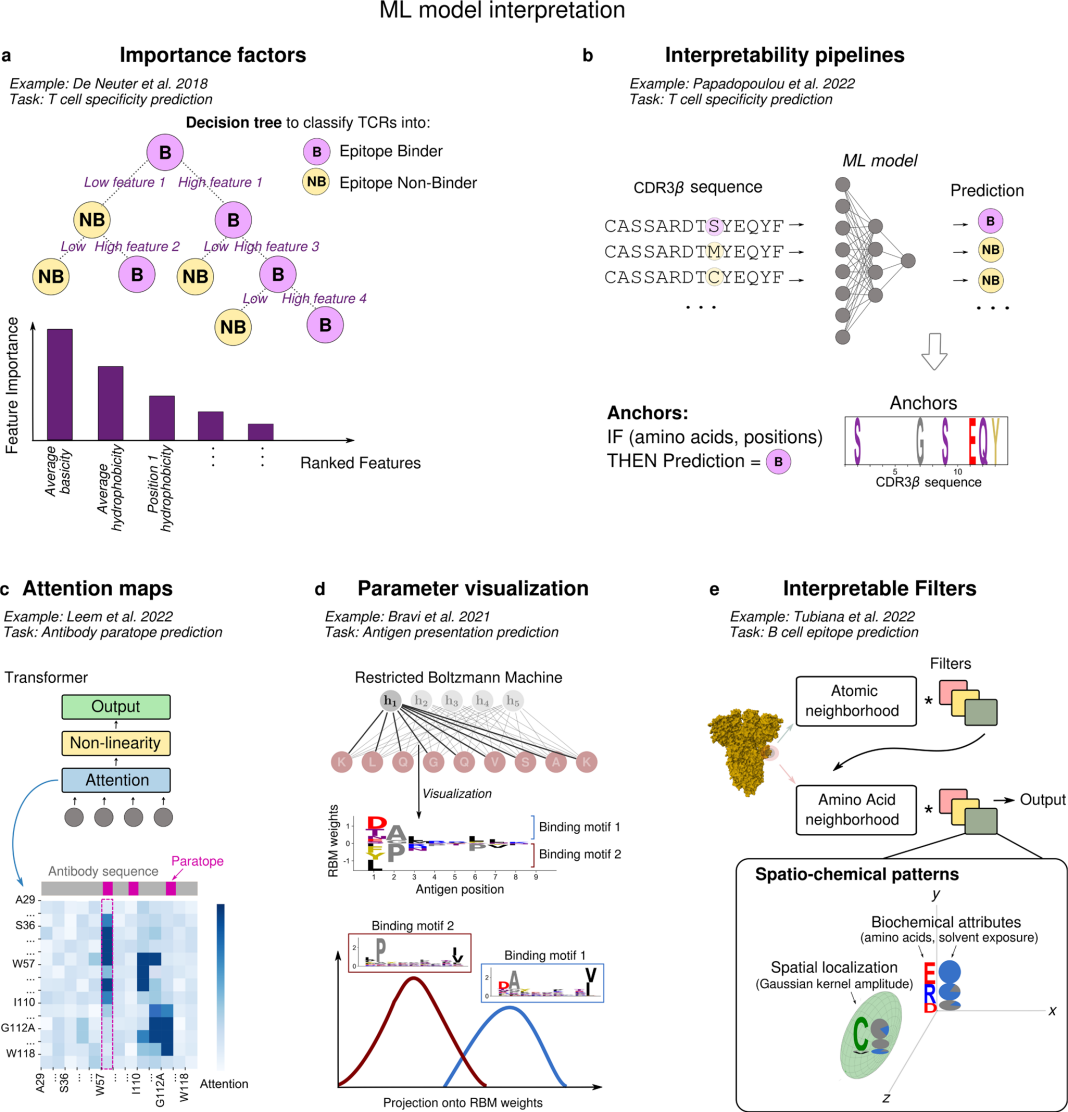
Strategies of ML model interpretability. **a** Feature importance; **b** interpretability pipelines; **c** attention maps; **d** weights visualization; **e** learnable spatio-chemical filters.

The need to better understand the molecular basis of epitope immunogenicity and epitope-paratope binding specificity highlights the importance to be able to extract biological insights from ML models. ML approaches that are explainable in terms of biological modes of action are increasingly recognized as a priority in immunology^154,155^, and more generally for ML applications of biomedical and clinical relevance^156^. Figure 4 introduces, in the form of graphical sketches, examples of ways in which the predictions from ML models can be made biologically interpretable, and how they have been employed in epitope discovery and epitope-paratope interaction studies.

Decision trees (Box 3) have been used for a variety of predictions relevant to immunology^30,101,149,157^, including classifying TCRs into specific binders of an epitope or non-binders^129,150,151^. The model ‘decides’ whether a TCR is binder or non-binder through a series of splits in the space of sequence features (e.g., average and positional physico-chemical properties), which are determined by whether a given feature is higher or lower than a threshold. Such decision rules take into account one feature at a time, hence the importance of each feature to the final prediction can be evaluated (Fig. 4a). Based on this analysis, ref. ^150^ finds that the average basicity of the CDR3 (on the *β* chain), as well as basicity and hydrophobicity of the amino acids in the CDR3*β* center, play an important role at discriminating epitope-specific from unspecific sequences.

Explainable predictions can be obtained in a model-agnostic fashion by applying interpretability pipelines, for example the one estimating ‘anchors’^158^. An anchor is an explanation that ‘anchors’ the model’s prediction locally to specific data attributes and formulated as an if-then rule. Once applied to models for classification of TCRs by epitope specificity^159^, anchors recapitulate the presence of specific amino acids in certain positions of epitope-specific TCR sequences (Fig. 4b), for example polar amino acids like serine (S) in the CDR3*β* binding to the peptide KLGGALQAK^159^.

The attention mechanism typical of transformers has been increasingly explored as tool to gain interpretability in protein language models^160^ (Fig. 4c, Box 3). For each sequence, an attention map describes how relevant each residue on the horizontal axis is in the prediction of all the other residues (vertical axis), detecting in this way structurally and functionally important residues that exhibit correlations with the other ones. In transformer models of antibody sequences, attention concentrates on sites in contact or belonging to the paratope^123^ (Fig. 4c).

ML architectures with a limited amount of parameters are more amenable to the inspection of the biological information learnt, for instance by direct visualization of their parameters. A point in case is the RBM architecture used to predict antigen presentation in ref. ^89^, whose main parameters are the sets of weights connecting the input layer to the only hidden layer (Box 3, Fig. 4d). The visualization of weights entering one hidden unit highlighted the existence of two distinct binding motifs within antigens of the same HLA type^89^, which correspond to two alternative and structurally validated HLA-binding modes^81^. Antigens bearing the different binding motifs can be readily distinguished via the projection of the data onto this set of weights (it is indeed this projection onto one or more sets of weights that defines the coordinates of the model’s representation space, where antigens cluster by sequence motifs connected to their HLA binding properties, see Fig. 2b).

Finally, so-called ‘geometric’ deep learning^161^ is another approach with potential for interpretability, as it models the underlying regularities of the data and leverages them for prediction. The protein binding site prediction method in ref. ^35^ implements this approach through the convolution (Box 3) of geometric representations of protein regions at the atomic and amino acid scale with learnable filters. Visualizing the parts of these representations that most contribute to each filter’s output results in interpretable spatio-chemical patterns, defined by sets of biochemical attributes (like specific amino acids and their degree of solvent exposure) along with their spatial coordinate (Fig. 4e). Such patterns highlight co-determinants of protein-protein binding like coordination number and electrostatic potential, generating insight also into the physico-chemical principles underlying antibody-epitope binding. For instance, ref. ^35^ detects a pattern at the amino acid scale positively correlated with epitope probability consisting of an exposed, charged amino acid close to a disulfide bond: this is a structural motif that confers stability, and hence plausibly facilitates high-affinity antibody binding.

These examples show that there exist interpretability strategies that are model-specific, relying on specific building blocks of a given ML architecture (like attention maps, Fig. 4c), and model-agnostic ones (like anchors, Fig. 4b), which are more broadly applicable to ML models to explain their output. In all cases, domain expertise has been essential to assess the biological relevance of the patterns learnt from immunological data. Strategies like the ones discussed, combined to domain expertise, point towards the feasibility of intepretable ML for molecular biology, and provide the basis for further work in this direction.

## Computational and ML tools in vaccine design beyond epitope prediction

Epitope identification is the most important prediction in rational vaccine design, yet it is only the starting point of the elaborate and challenging process of vaccine design (Fig. 1a). After epitope prediction has returned a set of vaccine candidates, additional computational methods and analyses are needed, first of all for the evaluation of structural and functional features of the candidate targets. This might further inform their selection and optimization along with the ML-enabled prediction of epitope-paratope interactions already discussed. Such evaluation steps (see refs. ^13,15,162–164^ for examples) consist of: structural modeling (e.g. with the tool^165^), to ensure surface accessibility of the predicted epitopes; molecular docking and molecular dynamics (e.g. with the tool^166^), to probe the stability and affinity of the binding between vaccine targets and immune receptors; screening of the targets’ similarity to the host proteome and of allergenicity (e.g. with the tools^167,168^), to discard the targets that can potentially trigger auto-immune reactions and side effects; evaluation of population coverage of the selected epitopes (e.g. with the tool^169^), as well as their degree of conservation, since targeting conserved regions might increase cross-variant protection; an assessment of biochemical properties such as solubility (e.g. with the tools^170,171^) that are key to the delivery and molecular mode of action of the selected targets; computer simulations of the immune response elicitable (e.g. with the tool^172^), to optimize vaccine dosage, formulation, and schedule. (The analysis resource section of IEDB^6^ makes available a number of computational tools for these tasks). ML is emerging as a technology that can assist also several of the evaluation steps, starting from structural characterization through the ground-breaking new ML methods for protein structure prediction^65–69^, as mentioned above. ML is increasingly used in drug design to predict computationally a number of molecular properties (for example solubility^173,174^); as such, it can guide the selection of adjuvants in vaccine construction^164,175^ or help predict mRNA stability to optimize mRNA-based vaccines’ intracellular delivery^176^.

Immune simulation approaches are moving toward combining the digital twins technology with ML^177^, an arena where ML can serve to incorporate proficiently pharmacokinetic and molecular binding data in the digital twin’s parameters to calibrate. In silico clinical trials^178^ are another set of computational models and simulation techniques to assist the assessment of safety and efficacy profiles in vaccine design. It is increasingly recognized that in silico clinical trials can be empowered by ML for tasks like: data augmentation (by generating synthetic patients to complement small-size cohorts^178^); outcome and response prediction^156^ (by detecting patterns in electronic records on previous trials and harnessing them for prediction); automation and optimization of participant recruitment, data collection and management, and trial monitoring^156,179,180^. These example tasks illustrate how ML could inform the design and planning of actual clinical trials to help improve their feasibility, efficiency, and success rate, albeit more work is needed for the large-scale deployment of such techniques.

Finally, a complementary and much needed scope of use for ML is to predict regions of the viral genome prone to harbor mutations, in such a way as to anticipate new variants before they emerge and design vaccine strategies robust to them. Approaches combining mathematical modeling and statistical learning have been developed to detect high-mutability regions^181,182^ and to model the fitness gain and potential for immune evasion conferred by mutations^183,184^. ML will contribute to boost their accuracy and applicability, by enhancing our understanding of epitope determinants in protein structure and sequence space and of the impact of mutations on epitope-paratope interactions.

## Limitations, challenges, and perspectives

In this perspective, I have discussed the type of predictions and methods by which ML can inform and guide vaccine target selection, mainly the tasks of B and T cell epitope discovery and the prediction of epitope-paratope interactions. There is a series of limitations and challenges, both at the level of datasets and methodology, that, once overcome, could pave the way to the wide application in rational vaccine design of the latest developments in this field.

### Data availability and quality

A key aspect to consider is that type, quality, and quantity of training data are crucial to the predictive power of any ML approach.

The main bottleneck preventing major leaps forward in the predictive performance of both structure- and sequence-based epitope-paratope interaction models is the scarcity of data to use as training sets. Training data should be seen as realizing a sampling of the full space of sequences and structures to model, and this sampling should be ideally exhaustive, or at least representative, of the modeled space and consistent. Major challenges are the extreme diversity of both the epitope and paratope sequence spaces to sample^185,186^, the cross-reactivity of epitope-paratope interactions, especially for T cells^187,188^, and their conformational diversity, with multiple binding modes even for the same target^189^. Assays sampling epitope-paratope binding pairs in a high-throughput fashion are lacking, already at the sequence level, and the available sequence data often consist only of a single chain. Available structural data, as mentioned, are even sparser. By way of example, solved structures of antibodies to date amount to a few thousands (7967 on the SabDab database^26^) and the ones of TCRs to a few hundreds (605 on the STCRDab database^190^). Ad hoc ML strategies can mitigate in part the problem of scarcity of data on epitope-paratope interactions by modeling them as particular instances of general protein-protein interactions, for which more data are available. Based on this assumption, a ML strategy pursued is to pre-train a model on these data, and next to fine-tune the model’s parameters on the epitope-paratope datasets^134,147^.

In addition to the limited amount of data, there is the problem that the aggregation of data from heterogeneous experimental assays can become a noise source, and the one of sampling biases. Antigens of biomedical interest that give rise to positive responses tend to be over-represented^111,191^, making it difficult to label with confidence ‘negative’ examples^36,192^; such a redundancy at the antigen level leads to models that are prone to overfitting and with imbalanced performance across epitopes (and concomitantly HLA types). Biases in the data can further propagate when new targets tested are chosen based on predictors trained on biased data, as it has been discussed in relation to peptide-HLA binding affinity assays^193^. On the other hand, mass spectrometry techniques used to map HLA-bound peptides suffer from technical biases in the detection of some amino acids, e.g. cysteine^19,193^.

An area requiring a concerted effort of the immunology community is thus the production, curation, and dissemination to ML experts of high-quality and internally consistent datasets. Efforts of method development need as well to be cognizant of existing biases, for example by including corrections for biased amino acid detection by mass spectrometry to improve performance for cystein-containing peptides^89^. Another promising avenue to resolve the lack of truly negative examples is to rely on ML approaches trained on positives vs unlabeled examples^36^ or positives only^106,119,120,146^. Bayesian inference has also been proposed to take into account biases and uncertainty in database annotation on T cell epitope immunogenicity and include systematically information on the number of responders to a given epitope^194^. Given the importance of the training data in setting performance, methods should be designed in such a way as to make re-training on newly produced data feasible and straightforward.

### Prediction performance and method integration

The advent of ML algorithms for the tasks discussed has led to better performance compared to more traditional bioinformatic approaches, yet there is still substantial room for improvement.

Controlled comparisons carried out in the literature are helping elucidate the entity of the improvements brought along by ML over bioinformatic approaches based on motifs, sequence similarity, or selected biophysical properties. The rather simple, linear motifs describing peptide-HLA-I binding preferences are well characterized by matrix-based models scoring independently every peptide position^79,81^, which have then comparable performance to neural network methods at scoring HLA-I presentation^18,19,88^; relatedly, the later methods tend to rely on shallow networks (typically limited to one hidden layer). To predict the immunogenicity of HLA-I-presented epitopes, we found that ML tools perform better than matrix-based ones, but also in this case the optimal predictor of immunogenicity is given by a shallow, as opposed to a deep, network^106^. ML methods give the best performance at predicting TCR specificity to HLA-I epitopes, but the difference compared to predictions based on TCR sequence similarity alone is rather modest^115^. On the other hand, a deep architecture is seen to have evident advantage over shallow and matrix-based models when predicting scores for HLA-II-presented epitopes^195^. Also for conformational B cell epitope prediction, large gains have been reported recently thanks to deep learning^35,36^, for instance compared to naive predictors scoring residues based on relative surface accessibility^36^. Hence, the need for training deep architectures, which enable to model highly non-linear input-output relationships but are data-demanding, is more or less clear depending on the prediction task. To bring clarity in this regard, regular, systematic benchmarks of the available methods on independent datasets and according to uniform assessment criteria are pivotal (see for example ref. ^115^), to recognize strengths and limitations in performance and to formulate recommendations for the next developments. IEDB performs automatic benchmarks of new predictors of HLA-I and II antigen presentation on the data that become available, in order to recommend methods and metrics for prediction, a procedure that, despite its pitfalls^19^, could serve as an example to follow.

One of the crucial problems performance-wise is the low precision of the final epitope identification, due to false positives, which can slow down and hamper the downstream steps of in silico, in vitro, and in vivo validation. For B cell epitopes, state-of-the-art methods^35,36^ assign to epitopes on average a score higher than ~ 70% of the scores for the same protein, indicating that many false positives do occur among the highest-ranked epitope residues. For class I T cell epitopes with well-characterized HLAs, with the best performing methods^88^ one has > 99% chance of identifying a presented antigen taking the top scoring peptide among all the possible ones from the proteome of interest; it is rather the subsequent prediction of immunogenic antigens among the presented ones that suffers from low precision, as reported in benchmarks with experimentally tested targets^21^.

The prediction of immunogenicity of candidate targets is particularly challenging, and ultimately can be validated only by experimental tests and clinical trials, being it an intrinsically multifactorial and multiscale effect. Firstly, protein-protein interactions are dynamic and susceptible to the cellular environment; a first step to account for these aspects is to complement ML predictions by molecular docking simulations of the interactions mediating the adaptive response (peptide docking to the HLA^57,196^, docking of TCRs to the peptide-HLA complex^56^, antigen-antibody docking^54,55^). Furthermore, immune activation and effector function are dependent on co-stimulatory signals, and more generally on the context at the cell and tissue level. For example, high antigen expression levels have been suggested to compensate for weak HLA-antigen binding, thus including cell type and tissue-specific information on antigen abundance has resulted in improved predictions of T cell epitopes^92,195,197^. Protection eventually depends on many factors, like innate control, infective dose, as well as the genetic and environmental factors that shape the individual immune repertoires (age, previous exposure, etc.). ML predictions should be therefore interpreted as inherently probabilistic, i.e., they come with an uncertainty stemming from the variety of factors that contribute to a positive response and are not included in the models.

Suboptimal precision implies more permissive prediction thresholds to ensure that a sufficient number of true epitopes is recovered, hence epitope prediction can result in hundreds of candidates to analyze and test, while only 10-30 subunits are necessary for the final construction of a multi-epitope vaccine^13,15^. Post-epitope prediction evaluations can be time-consuming for this reason, and because usually their steps are not integrated and automated. A strategy for higher-efficiency vaccine design recently proposed^15^ compresses epitope identification and property evaluation in one step by training a deep neural network to directly predict vaccine subunits with the desired properties, which results in fewer candidates to further evaluate. Indeed, more rapid screening of possible targets requires the development of frameworks that can perform and combine multiple predictions, similarly to approaches in drug discovery integrating ML models and docking simulations^198^. The design of such target selection pipelines integrating different prediction steps will benefit from ML methods that are clearly documented in terms of scope, modes, and optimal conditions of use. Working with standardized input formats and output metrics would be also important to save efforts of data pre-processing and post-processing and to facilitate method integration. For the future development of rational vaccine technologies, two of the most pressing needs are hence: increased precision of epitope prediction, to reliably narrow down target selection to fewer candidates; integrated frameworks connecting the bioinformatic and ML software necessary for ML-assisted epitope prediction and the subsequent evaluations, possibly developed within a user-friendly infrastructure that is easy to access and implement either as a web server or a downloadable package. Such improvements are essential to reducing the time, manual work, and resources involved in vaccine target selection and validation, thus they are prerequisites to the flexibility and scalability sought-after in personalized neoantigen discovery and in the adaptation of vaccines to newly-emerged viral strains.

## Data Availability

No datasets were generated or analyzed in this article.

## Code availability

No new algorithms or code were developed or used for this article.

## References

[1] He L, Zhu J (2015). Computational tools for epitope vaccine design and evaluation. Curr. Opin. Virol..

[2] Sette A, Rappuoli R (2010). Reverse vaccinology: developing vaccines in the era of genomics. Immunity.

[3] Kyriakidis, N. C. et al. SARS-CoV-2 vaccines strategies: a comprehensive review of phase 3 candidates. *npj Vaccines* **6**, 1–17 (2021). 10.1038/s41541-021-00292-w PMC7900244 33619260

[4] Soria-Guerra RE, Nieto-Gomez R, Govea-Alonso DO, Rosales-Mendoza S (2015). An overview of bioinformatics tools for epitope prediction: implications on vaccine development. J. Biomed. Inform..

[5] Srivastava, S., Chatziefthymiou, S. D. & Kolbe, M. Vaccines Targeting Numerous Coronavirus Antigens, Ensuring Broader Global Population Coverage: Multi-epitope and Multi-patch Vaccines. In *Vaccine Design: Methods and Protocols, Volume 1. Vaccines for Human Diseases. Methods in Molecular Biology*. (ed. Thomas, S.) 149–175 (Springer US, 2022). 10.1007/978-1-0716-1884-4_7 34914046

[6] Vita R (2019). The immune epitope database (IEDB): 2018 update. Nucleic Acids Res..

[7] Dimitrov I, Zaharieva N, Doytchinova I (2020). Bacterial immunogenicity prediction by machine learning methods. Vaccines.

[8] Ong E (2021). Vaxign2: the second generation of the first web-based vaccine design program using reverse vaccinology and machine learning. Nucleic Acids Res..

[9] Herrera-Bravo J (2021). VirVACPRED: a web server for prediction of protective viral antigens. Int. J. Pept. Res. Ther..

[10] Bowman BN (2011). Improving reverse vaccinology with a machine learning approach. Vaccine.

[11] Heinson AI (2017). Enhancing the biological relevance of machine learning classifiers for reverse vaccinology. Int. J. Mol. Sci..

[12] Ong E (2020). Vaxign-ML: supervised machine learning reverse vaccinology model for improved prediction of bacterial protective antigens. Bioinformatics.

[13] Ong, E., Wong, MU., Huffman, A. & He, Y. COVID-19 coronavirus vaccine design using reverse vaccinology and machine learning. *Front. Immunol.* **11**, 1581 (2020). 10.3389/fimmu.2020.01581 PMC7350702 32719684

[14] Yarmarkovich M, Warrington JM, Farrel A, Maris JM (2020). Identification of SARS-CoV-2 vaccine epitopes predicted to induce long-term population-scale immunity. Cell Rep. Med..

[15] Yang Z, Bogdan P, Nazarian S (2021). An in silico deep learning approach to multi-epitope vaccine design: A SARS-CoV-2 case study. Sci. Rep..

[16] Mohanty E, Mohanty A (2021). Role of artificial intelligence in peptide vaccine design against RNA Viruses. Inf. Med. Unlocked.

[17] Swadling L (2022). Pre-existing polymerase-specific T cells expand in abortive seronegative SARS-CoV-2. Nature.

[18] Mei S (2019). A comprehensive review and performance evaluation of bioinformatics tools for HLA class I peptide-binding prediction. Brief. Bioinform..

[19] Nielsen M, Andreatta M, Peters B, Buus S (2020). Immunoinformatics: predicting peptide–MHC binding. Annu. Rev. Biomed. Data Sci..

[20] Kar, P., Ruiz-Perez, L., Arooj, M. & Mancera, R. L. Current methods for the prediction of T-cell epitopes. *Pept. Sci.* **110**, e24046 (2018).

[21] Buckley PR (2022). Evaluating performance of existing computational models in predicting CD8+ T cell pathogenic epitopes and cancer neoantigens. Brief. Bioinform..

[22] Lee CH (2020). Predicting cross-reactivity and antigen specificity of T cell receptors. Front. Immunol..

[23] Norman RA (2020). Computational approaches to therapeutic antibody design: established methods and emerging trends. Brief. Bioinform..

[24] Kim J, McFee M, Fang Q, Abdin O, Kim PM (2023). Computational and artificial intelligence-based methods for antibody development. Trends Pharmacol. Sci..

[25] Shugay M (2018). VDJdb: a curated database of t-cell receptor sequences with known antigen specificity. Nucleic Acids Res..

[26] Dunbar J (2014). SAbDab: the structural antibody database. Nucleic Acids Res..

[27] Saha S, Raghava GPS (2006). Prediction of continuous B-cell epitopes in an antigen using recurrent neural network. Proteins.

[28] Rubinstein ND, Mayrose I, Pupko T (2009). A machine-learning approach for predicting B-cell epitopes. Mol. Immunol..

[29] Zhao L, Wong L, Lu L, Hoi SC, Li J (2012). B-cell epitope prediction through a graph model. BMC Bioinform..

[30] Jespersen MC, Peters B, Nielsen M, Marcatili P (2017). BepiPred-2.0: improving sequence-based B-cell epitope prediction using conformational epitopes. Nucleic Acids Res..

[31] Clifford JN (2022). BepiPred-3.0: improved B-cell epitope prediction using protein language models. Protein Sci.: Publ. Protein Soc..

[32] Liu T, Shi K, Li W (2020). Deep learning methods improve linear B-cell epitope prediction. BioData Mining.

[33] da Silva BM, Myung Y, Ascher DB, Pires DEV (2022). epitope3D: a machine learning method for conformational B-cell epitope prediction. Brief. Bioinform..

[34] Shashkova, T. I. et al. SEMA: antigen B-cell conformational epitope prediction using deep transfer learning. *Front. Immunol.* **13**, 960985 (2022). 10.3389/fimmu.2022.960985 PMC9523212 36189325

[35] Tubiana, J., Schneidman-Duhovny, D. & Wolfson, H. J. ScanNet: an interpretable geometric deep learning model for structure-based protein binding site prediction. *Nat. Methods* **19**, 730–739 (2022). 10.1038/s41592-022-01490-7 35637310

[36] Høie, M. H. et al. DiscoTope-3.0 - improved B-celL epitope prediction using AlphaFold2 modeling and inverse folding latent representations. *bioRxiv*10.1101/2023.02.05.527174 (2023).

[37] Parker JM, Guo D, Hodges RS (1986). New hydrophilicity scale derived from high-performance liquid chromatography peptide retention data: correlation of predicted surface residues with antigenicity and X-ray-derived accessible sites. Biochemistry.

[38] Kolaskar AS, Tongaonkar PC (1990). A semi-empirical method for prediction of antigenic determinants on protein antigens. FEBS Lett..

[39] Karplus PA, Schulz GE (1985). Prediction of chain flexibility in proteins. Naturwissenschaften.

[40] Thornton JM, Edwards MS, Taylor WR, Barlow DJ (1986). Location of ’continuous’ antigenic determinants in the protruding regions of proteins. EMBO J..

[41] Ponomarenko J (2008). ElliPro: a new structure-based tool for the prediction of antibody epitopes. BMC Bioinform..

[42] Emini EA, Hughes JV, Perlow DS, Boger J (1985). Induction of hepatitis A virus-neutralizing antibody by a virus-specific synthetic peptide. J. Virol..

[43] Ingraham, J., Garg, V. K., Barzilay, R. & Jaakkola, T. *Generative Models for Graph-Based Protein Design. NIPS 2019* (2019).

[44] Strokach, A., Becerra, D., Corbi-Verge, C. & Kim, P. M. Fast and flexible protein design using deep graph neural networks. *Cell Syst.* **11**, 402–411.e4 (2020). 10.1016/j.cels.2020.08.016 32971019

[45] Fout, A., Byrd, J., Shariat, B. & Ben-Hur A. Protein interface prediction using graph convolutional networks. In: *Advances in Neural Information Processing Systems*. vol. 30 (Curran Associates, Inc., 2017).

[46] Yuan Q, Chen J, Zhao H, Zhou Y, Yang Y (2021). Structure-aware protein–protein interaction site prediction using deep graph convolutional network. Bioinformatics.

[47] Abdollahi, N., Tonekaboni, S. A. M., Huang, J., Wang, B. & MacKinnon, S. NodeCoder: a graph-based machine learning platform to predict active sites of modeled protein structures. *arXiv*10.48550/arXiv.2302.03590 (2023).

[48] Cha M (2022). Unifying structural descriptors for biological and bioinspired nanoscale complexes. Nat. Comput. Sci..

[49] Roche R, Moussad B, Shuvo MH, Bhattacharya D (2023). E(3) equivariant graph neural networks for robust and accurate protein-protein interaction site prediction. PLoS Comput. Biol..

[50] Ferreira, M. V., Nogueira, T., Rios, R. A., Lopes, T. J. S. A graph-based machine learning framework identifies critical properties of FVIII that lead to Hemophilia A. *Front. Bioinform*. **3**, 1152039 (2023). 10.3389/fbinf.2023.1152039 PMC10206133 37235045

[51] Zhou J (2020). Graph neural networks: a review of methods and applications. AI Open.

[52] Hsu, C. et al. Learning inverse folding from millions of predicted structures. In: *Proceedings of the 39th International Conference on Machine Learning*. p. 8946–8970 (PMLR, 2022).

[53] Muhammed MT, Aki-Yalcin E (2019). Homology modeling in drug discovery: overview, current applications, and future perspectives. Chem. Biol. Drug Des..

[54] Ambrosetti F, Jiménez-García B, Roel-Touris J, Bonvin AMJJ (2020). Modeling antibody-antigen complexes by information-driven docking. Structure.

[55] Schoeder, C. T. et al. Modeling immunity with rosetta: methods for antibody and antigen design. *Biochemistry* **60**, 825–846 (2021). 10.1021/acs.biochem.0c00912 PMC7992133 33705117

[56] Peacock, T. & Chain, B. Information-driven docking for TCR-pMHC complex prediction. *Front. Immunol.* **12**, 686127 (2021). 10.3389/fimmu.2021.686127 PMC8219952 34177934

[57] Atanasova M, Doytchinova I (2023). Docking-based prediction of peptide binding to MHC proteins. Methods Mol. Biol..

[58] Dormitzer PR, Ulmer JB, Rappuoli R (2008). Structure-based antigen design: a strategy for next generation vaccines. Trends Biotechnol..

[59] Higgins, M. K. Can we AlphaFold our way out of the next pandemic? *J. Mol. Biol.* **433**, 167093 (2021). 10.1016/j.jmb.2021.167093 PMC8186955 34116123

[60] Pavlova A (2021). Machine learning reveals the critical interactions for SARS-CoV-2 spike protein binding to ACE2. J. Phys. Chem. Lett..

[61] Benevenuta S, Pancotti C, Fariselli P, Birolo G, Sanavia T (2021). An antisymmetric neural network to predict free energy changes in protein variants. J. Phys. D: Appl. Phys..

[62] Li B, Yang YT, Capra JA, Gerstein MB (2020). Predicting changes in protein thermodynamic stability upon point mutation with deep 3D convolutional neural networks. PLoS Comput. Biol..

[63] Pucci F, Schwersensky M, Rooman M (2022). Artificial intelligence challenges for predicting the impact of mutations on protein stability. Curr. Opin. Struct. Biol..

[64] Dauparas J (2022). Robust deep learning–based protein sequence design using proteinMPNN. Science.

[65] Senior AW (2020). Improved protein structure prediction using potentials from deep learning. Nature.

[66] Jumper J (2021). Highly accurate protein structure prediction with AlphaFold. Nature.

[67] Evans, R. et al. Protein complex prediction with AlphaFold-Multimer. *biorxiv*10.1101/2021.10.04.463034 (2022).

[68] Du Z (2021). The trRosetta server for fast and accurate protein structure prediction. Nat. Protoc..

[69] Baek M (2021). Accurate prediction of protein structures and interactions using a 3-track neural network. Science.

[70] Hederman AP, Ackerman ME (2023). Leveraging deep learning to improve vaccine design. Trends Immunol..

[71] Ruffolo JA, Guerra C, Mahajan SP, Sulam J, Gray JJ (2020). Geometric potentials from deep learning improve prediction of CDR H3 loop structures. Bioinformatics.

[72] Ruffolo JA, Sulam J, Gray JJ (2022). Antibody structure prediction using interpretable deep learning. Patterns.

[73] Abanades B, Georges G, Bujotzek A, Deane CM (2022). ABlooper: fast accurate antibody cdr loop structure prediction with accuracy estimation. Bioinformatics.

[74] Ruffolo JA, Chu LS, Mahajan SP, Gray JJ (2023). Fast, accurate antibody structure prediction from deep learning on massive set of natural antibodies. Nat. Commun..

[75] Bradley P (2023). Structure-based prediction of T cell receptor: peptide-MHC interactions. eLife.

[76] Chinery L, Wahome N, Moal I, Deane CM (2023). Paragraph—antibody paratope prediction using graph neural networks with minimal feature vectors. Bioinformatics.

[77] Grifoni A (2020). A sequence homology and bioinformatic approach can predict candidate targets for immune responses to SARS-CoV-2. Cell Host Microbe.

[78] Vitiello A, Zanetti M (2017). Neoantigen prediction and the need for validation. Nat. Biotechnol..

[79] Bassani-Sternberg M, Gfeller D (2016). Unsupervised HLA peptidome deconvolution improves ligand prediction accuracy and predicts cooperative effects in peptide–HLA interactions. J. Immunol..

[80] Bassani-Sternberg M (2017). Deciphering HLA-I motifs across HLA peptidomes improves neo-antigen predictions and identifies allostery regulating HLA specificity. PLoS Comput. Biol..

[81] Gfeller D (2018). The length distribution and multiple specificity of naturally presented HLA-I ligands. J. Immunol..

[82] O’Donnell TJ (2018). MHCflurry: open-source class I MHC binding affinity prediction. Cell Syst..

[83] O’Donnell TJ, Rubinsteyn A, Laserson U (2020). MHCflurry 2.0: improved pan-allele prediction of MHC class I-presented peptides by incorporating antigen processing. Cell Syst..

[84] Nielsen M (2007). NetMHCpan, a method for quantitative predictions of peptide binding to any HLA-A and -B locus protein of known sequence. PLoS ONE.

[85] Lundegaard, C. et al. NetMHC-3.0: accurate web accessible predictions of human, mouse and monkey MHC Class I affinities for peptides of length 8–11. *Nucleic Acids Res*. **36**, W509–W512 (2008). 10.1093/nar/gkn202 PMC2447772 18463140

[86] Andreatta M, Nielsen M (2016). Gapped sequence alignment using artificial neural networks: application to the MHC class I system. Bioinformatics.

[87] Jurtz V (2017). NetMHCpan-4.0: improved peptide–MHC class I interaction predictions integrating eluted ligand and peptide binding affinity data. J. Immunol..

[88] Reynisson B, Alvarez B, Paul S, Peters B, Nielsen M (2020). NetMHCpan-4.1 and NetMHCIIpan-4.0: improved predictions of MHC antigen presentation by concurrent motif deconvolution and integration of MS MHC eluted ligand data. Nucleic Acids Res..

[89] Bravi B (2021). RBM-MHC: a semi-supervised machine-learning method for sample-specific prediction of antigen presentation by HLA-I alleles. Cell Syst..

[90] Abelin JG (2017). Mass spectrometry profiling of HLA-associated peptidomes in mono-allelic cells enables more accurate epitope prediction. Immunity.

[91] Abelin, JG. et al. Defining HLA-II ligand processing and binding rules with mass spectrometry enhances cancer epitope prediction. *Immunity* **51**, 766–779.e17 (2019). 10.1016/j.immuni.2019.08.012 31495665

[92] Sarkizova S (2020). A large peptidome dataset improves HLA class I epitope prediction across most of the human population. Nat. Biotechnol..

[93] Racle J (2019). Robust prediction of HLA class II epitopes by deep motif deconvolution of immunopeptidomes. Nat. Biotechnol..

[94] Lawrence PJ, Ning X (2022). Improving MHC class I antigen-processing predictions using representation learning and cleavage site-specific kernels. Cell Rep. Methods.

[95] Reynisson B (2020). Improved prediction of MHC II antigen presentation through integration and motif deconvolution of mass spectrometry MHC eluted ligand data. J. Proteome Res..

[96] Racle J (2023). Machine learning predictions of MHC-II specificities reveal alternative binding mode of class II epitopes. Immunity.

[97] Nilsson JB (2023). Machine learning reveals limited contribution of trans-only encoded variants to the HLA-DQ immunopeptidome. Commun. Biol..

[98] Calis JJA (2013). Properties of MHC class I presented peptides that enhance immunogenicity. PLoS Comput. Biol..

[99] Trolle T, Nielsen M (2014). NetTepi: an integrated method for the prediction of T cell epitopes. Immunogenetics.

[100] Chowell D (2015). TCR contact residue hydrophobicity is a hallmark of immunogenic CD8+ T cell epitopes. Proc. Natl Acad. Sci. USA.

[101] Ogishi M, Yotsuyanagi H (2019). Quantitative prediction of the landscape of T cell epitope immunogenicity in sequence space. Front. Immunol..

[102] Riley TP (2019). Structure based prediction of neoantigen immunogenicity. Front. Immunol..

[103] Schmidt J (2021). Prediction of neo-epitope immunogenicity reveals TCR recognition determinants and provides insight into immunoediting. Cell Rep. Med..

[104] Gfeller D (2023). Improved predictions of antigen presentation and TCR recognition with MixMHCpred2.2 and PRIME2.0 reveal potent SARS-CoV-2 CD8+ T-cell epitopes. Cell Syst..

[105] Dhanda SK (2018). Predicting HLA CD4 immunogenicity in human populations. Front. Immunol..

[106] Bravi, B. et al. A transfer-learning approach to predict antigen immunogenicity and T-cell receptor specificity. *eLife* **12**, e85126 (2023). 10.7554/eLife.85126 PMC10522340 37681658

[107] Finotello F, Rieder D, Hackl H, Trajanoski Z (2019). Next-generation computational tools for interrogating cancer immunity. Nat. Rev. Genet..

[108] Roudko V, Greenbaum B, Bhardwaj N (2020). Computational prediction and validation of tumor-associated neoantigens. Front. Immunol..

[109] Roesler AS, Anderson KS (2022). Beyond sequencing: prioritizing and delivering neoantigens for cancer vaccines. Methods Mol. Biol..

[110] Wells, D. K. et al. Key parameters of tumor epitope immunogenicity revealed through a consortium approach improve neoantigen prediction. *Cell* **183**, 818–834.e13 (2020). 10.1016/j.cell.2020.09.015 PMC7652061 33038342

[111] Schaap-Johansen AL, Vujović M, Borch A, Hadrup SR, Marcatili P (2021). T cell epitope prediction and its application to immunotherapy. Front. Immunol..

[112] Croft NP (2019). Most viral peptides displayed by class I MHC on infected cells are immunogenic. Proc. Natl Acad. Sci. USA.

[113] Bjerregaard, A. M. et al. An analysis of natural T cell responses to predicted tumor neoepitopes. *Front. Immunol.* **8**, 1566 (2017). 10.3389/fimmu.2017.01566 PMC5694748 29187854

[114] Kristensen, N. P. et al. Neoantigen-reactive CD8^+^ T cells affect clinical outcome of adoptive cell therapy with tumor-infiltrating lymphocytes in melanoma. *J. Clin. Investig*. 132, e150535 (2022). 10.1172/JCI150535 PMC8759789 34813506

[115] Meysman, P. et al. Benchmarking solutions to the T-cell receptor epitope prediction problem: IMMREP22 workshop report. *ImmunoInformatics* **9**, 100024 (2023).

[116] Liberis E, Veličković P, Sormanni P, Vendruscolo M, Liò P (2018). Parapred: antibody paratope prediction using convolutional and recurrent neural networks. Bioinformatics.

[117] Ambrosetti F (2020). proABC-2: PRediction of AntiBody contacts v2 and its application to information-driven docking. Bioinformatics.

[118] Daberdaku S, Ferrari C (2019). Antibody interface prediction with 3D zernike descriptors and SVM. Bioinformatics.

[119] Isacchini, G., Walczak, A. M., Mora, T. & Nourmohammad, A. Deep generative selection models of T and B cell receptor repertoires with soNNia. *Proc. Natl Acad. Sci. USA*. **118**, e2023141118 (2021). 10.1073/pnas.2023141118 PMC8040596 33795515

[120] Bravi B (2021). Probing T-cell response by sequence-based probabilistic modeling. PLoS Comput. Biol..

[121] Wu, K. et al. TCR-BERT: learning the grammar of T-cell receptors for flexible antigen-binding analyses. *The 2022 ICML Workshop on Computational Biology* (2022).

[122] Sidhom JW, Larman HB, Pardoll DM, Baras AS (2021). DeepTCR is a deep learning framework for revealing sequence concepts within T-cell repertoires. Nat. Commun..

[123] Leem J, Mitchell LS, Farmery JHR, Barton J, Galson JD (2022). Deciphering the language of antibodies using self-supervised learning. Patterns.

[124] Akbar R (2022). In silico proof of principle of machine learning-based antibody design at unconstrained scale. mAbs.

[125] Saka K (2021). Antibody design using LSTM based deep generative model from phage display library for affinity maturation. Sci. Rep..

[126] Shin JE (2021). Protein design and variant prediction using autoregressive generative models. Nat. Commun..

[127] Jokinen E, Huuhtanen J, Mustjoki S, Heinonen M, Lähdesmäki H (2021). Predicting recognition between T cell receptors and epitopes with TCRGP. PLoS Comput. Biol..

[128] Zhang W (2021). A framework for highly multiplexed dextramer mapping and prediction of T cell receptor sequences to antigen specificity. Sci. Adv..

[129] Gielis, S. et al. Detection of enriched T cell epitope specificity in full T cell receptor sequence repertoires. *Front. Immunol.* **10**, 2820 (2019). 10.3389/fimmu.2019.02820 PMC6896208 31849987

[130] Croce, G. et al. Deep learning predictions of TCR-epitope interactions reveal epitope-specific chains in dual alpha T cells. *bioRxiv*10.1101/2023.09.13.557561 (2023). 10.1038/s41467-024-47461-8 PMC11016097 38615042

[131] Dash, P. et al. Quantifiable predictive features define epitope-specific T cell receptor repertoires. *Nature* **547**, 89–93 (2017). 10.1038/nature22383 PMC5616171 28636592

[132] Glanville J (2017). Identifying specificity groups in the T cell receptor repertoire. Nature.

[133] Mayer-Blackwell K (2021). TCR meta-clonotypes for biomarker discovery with Tcrdist3 Enabled identification of public, HLA-restricted clusters of SARS-CoV-2 TCRs. eLife.

[134] Weber A, Born J, Rodriguez Martínez M (2021). TITAN: T-cell receptor specificity prediction with bimodal attention networks. Bioinformatics.

[135] Moris P (2021). Current challenges for unseen-epitope TCR interaction prediction and a new perspective derived from image classification. Brief. Bioinform..

[136] Springer, I., Tickotsky, N. & Louzoun, Y. Contribution of T cell receptor alpha and beta CDR3, MHC typing, V and J genes to peptide binding prediction. *Front. Immunol*. **12**, 664514 (2021). 10.3389/fimmu.2021.664514 PMC8107833 33981311

[137] Lu T (2021). Deep learning-based prediction of the T cell receptor–antigen binding specificity. Nat. Mach. Intell..

[138] Xu Z (2021). DLpTCR: an ensemble deep learning framework for predicting immunogenic peptide recognized by T cell receptor. Brief. Bioinform..

[139] Grazioli F (2023). Attentive variational information bottleneck for TCR–peptide interaction prediction. Bioinformatics.

[140] Gao Y (2023). Pan-peptide meta learning for T-cell receptor–antigen binding recognition. Nat. Mach. Intell..

[141] Huang, Y., Zhang, Z. & Zhou, Y. AbAgIntPre: a deep learning method for predicting antibody-antigen interactions based on sequence information. *Front. Immunol*. **13**, 1053617 (2022). 10.3389/fimmu.2022.1053617 PMC9813736 36618397

[142] Schneider C, Buchanan A, Taddese B (2022). DLAB: deep learning methods for structure-based virtual screening of antibodies. Bioinformatics.

[143] Milighetti, M., Shawe-Taylor, J. & Chain, B. Predicting T cell receptor antigen specificity from structural features derived from homology models of receptor-peptide-major histocompatibility complexes. *Front. Physiol.* **12**, 730908 (2021). 10.3389/fphys.2021.730908 PMC8456106 34566692

[144] Montemurro A (2021). NetTCR-2.0 enables accurate prediction of TCR-peptide binding by using paired TCR*α* and *β* sequence data. Commun. Biol..

[145] Jensen, M. F. & Nielsen, M. NetTCR 2.2 - improved TCR specificity predictions by combining pan- and peptide-specific training strategies, loss-scaling and integration of sequence similarity. *bioRxiv*10.1101/2023.10.12.562001 (2023). 10.7554/eLife.93934 PMC10942633 38437160

[146] Meynard-Piganeau, B., Feinauer, C., Weigt, M., Walczak, A. M. & Mora, T. TULIP — a transformer based unsupervised language model for interacting peptides and T-cell receptors that generalizes to unseen epitopes. *bioRxiv*10.1101/2023.07.19.549669 (2023). 10.1073/pnas.2316401121 PMC11181096 38838016

[147] Pittala, S. & Bailey-Kellogg, C. Learning context-aware structural representations to predict antigen and antibody binding interfaces. *Bioinformatics* **36**, 3996–4003 (2020). 10.1093/bioinformatics/btaa263 PMC7332568 32321157

[148] Myung Y, Pires DEV, Ascher DB (2022). CSM-AB: graph-based antibody-antigen binding affinity prediction and docking scoring function. Bioinformatics.

[149] Yang YX, Wang P, Zhu BT (2023). Binding affinity prediction for antibody-protein antigen complexes: a machine learning analysis based on interface and surface areas. J. Mol. Graph. Model..

[150] De Neuter N (2018). On the feasibility of mining CD8+ T cell receptor patterns underlying immunogenic peptide recognition. Immunogenetics.

[151] Tong Y (2020). SETE: sequence-based ensemble learning approach for TCR epitope binding prediction. Comput. Biol. Chem..

[152] Rives A (2021). Biological structure and function emerge from scaling unsupervised learning to 250 million protein sequences. Proc. Natl Acad. Sci. USA.

[153] Bepler T, Berger B (2021). Learning the protein language: evolution, structure, and function. Cell Syst..

[154] Dens C, Bittremieux W, Affaticati F, Laukens K, Meysman P (2023). Interpretable deep learning to uncover the molecular binding patterns determining TCR–epitope interaction predictions. ImmunoInformatics.

[155] Rodríguez Martínez, M., Barberis, M. & Niarakis, A. Computational modelling of immunological mechanisms: from statistical approaches to interpretable machine learning. *ImmunoInformatics*. **12**, 100029 (2023).

[156] Askin S, Burkhalter D, Calado G (2023). Artificial intelligence applied to clinical trials: opportunities and challenges. Health Technol..

[157] Olimpieri PP, Chailyan A, Tramontano A, Marcatili P (2013). Prediction of site-specific interactions in antibody-antigen complexes: The proABC method and server. Bioinformatics.

[158] Ribeiro, M. T., Singh, S. & Guestrin, C. Anchors: high-precision model-agnostic explanations. *Proc. AAAI Conf. Artif. Intell*. **32** https://ojs.aaai.org/index.php/AAAI/article/view/11491 (2018).

[159] Papadopoulou I, Nguyen AP, Weber A, Martínez MR (2022). DECODE: a computational pipeline to discover T cell receptor binding rules. Bioinformatics.

[160] Vig, J. et al. BERTology meets biology: interpreting attention in protein language models. In *9th International Conference on Learning Representations (ICLR,* 2021).

[161] Bronstein, MM., Bruna, J., Cohen, T., Veličković, P. Geometric deep learning: grids, groups, graphs, geodesics, and gauges. *arXiv*10.48550/arXiv.2104.13478 (2021).

[162] Malone B (2020). Artificial intelligence predicts the immunogenic landscape of SARS-CoV-2 leading to universal blueprints for vaccine designs. Sci. Rep..

[163] Samad A (2022). Designing a multi-epitope vaccine against SARS-CoV-2: an immunoinformatics approach. J. Biomol. Struct. Dyn..

[164] Thomas S, Abraham A, Baldwin J, Piplani S, Petrovsky N (2022). Artificial intelligence in vaccine and drug design. Methods Mol. Biol..

[165] Källberg M (2012). Template-based protein structure modeling using the RaptorX web server. Nat. Protoc..

[166] Kozakov D (2017). The ClusPro web server for protein–protein docking. Nat. Protoc..

[167] Kim CK (2014). AllergenPro: an integrated database for allergenicity analysis and prediction. Bioinformation.

[168] Dimitrov I, Naneva L, Doytchinova I, Bangov I (2014). AllergenFP: allergenicity prediction by descriptor fingerprints. Bioinformatics.

[169] Bui HH (2006). Predicting population coverage of T-cell epitope-based diagnostics and vaccines. BMC Bioinform..

[170] Gasteiger, E. et al. Protein Identification and Analysis Tools on the ExPASy Server. In *The Proteomics Protocols Handbook. Springer Protocols Handbooks*. (ed. Walker, J. M.) 571–607 (Humana Press, 2005).

[171] Magnan CN, Randall A, Baldi P (2009). SOLpro: accurate sequence-based prediction of protein solubility. Bioinformatics.

[172] Rapin N, Lund O, Bernaschi M, Castiglione F (2010). Computational immunology meets bioinformatics: the use of prediction tools for molecular binding in the simulation of the immune system. PLoS ONE.

[173] Khurana S (2018). DeepSol: a deep learning framework for sequence-based protein solubility prediction. Bioinformatics.

[174] Ansari M, White AD (2023). Serverless prediction of peptide properties with recurrent neural networks. J. Chem. Inf. Model..

[175] Hioki, K. et al. Machine learning-assisted screening of herbal medicine extracts as vaccine adjuvants. *Front. Immunol*. **13**, 847616 (2022). 10.3389/fimmu.2022.847616 PMC9160479 35663999

[176] Wayment-Steele HK (2022). Deep learning models for predicting RNA degradation via dual crowdsourcing. Nat. Mach. Intell..

[177] Zohdi TI (2022). Machine-learning and digital-twins for rapid evaluation and design of injected vaccine immune-system responses. Comput. Methods Appl. Mech. Eng..

[178] Pappalardo F, Russo G, Tshinanu FM, Viceconti M (2019). In silico clinical trials: concepts and early adoptions. Brief. Bioinform..

[179] Chaudhari N, Ravi R, Gogtay NJ, Thatte UM (2020). Recruitment and retention of the participants in clinical trials: challenges and solutions. Perspect. Clin. Res..

[180] Weissler EH (2021). The role of machine learning in clinical research: transforming the future of evidence generation. Trials.

[181] Jain S, Xiao X, Bogdan P, Bruck J (2021). Generator based approach to analyze mutations in genomic datasets. Sci. Rep..

[182] Rodriguez-Rivas J, Croce G, Muscat M, Weigt M (2022). Epistatic models predict mutable sites in SARS-CoV-2 proteins and epitopes. Proc. Natl Acad. Sci. USA.

[183] Łuksza M, Lässig M (2014). A predictive fitness model for influenza. Nature.

[184] Barton, J. P. et al. Relative rate and location of intra-host HIV evolution to evade cellular immunity are predictable. *Nat. Commun.* **7**, 11660 (2016). 10.1038/ncomms11660 PMC4879252 27212475

[185] Paul S (2013). HLA class I alleles are associated with peptide-binding repertoires of different size, affinity, and immunogenicity. J. Immunol..

[186] Mora T, Walczak AM (2019). How many different clonotypes do immune repertoires contain. Curr. Opin. Syst. Biol..

[187] Mason D (1998). A very high level of crossreactivity is an essential feature of the T-cell receptor. Immunol. Today.

[188] Birnbaum ME (2014). Deconstructing the peptide-MHC specificity of T cell recognition. Cell.

[189] Bradley P, Thomas PG (2019). Using T cell receptor repertoires to understand the principles of adaptive immune recognition. Annu. Rev. Immunol..

[190] Leem, J., de Oliveira, S. H. P., Krawczyk, K. & Deane, C. M. STCRDab: the structural T-cell receptor database. *Nucleic Acids Res.* **46**, D406–D412 (2018). 10.1093/nar/gkx971 PMC5753249 29087479

[191] Hudson, D., Fernandes, RA., Basham, M., Ogg, G. & Koohy, H. Can we predict T cell specificity with digital biology and machine learning? *Nat. Rev. Immunol.* **23**, 1–11 (2023). 10.1038/s41577-023-00835-3 PMC9908307 36755161

[192] Dalsass, M., Brozzi, A., Medini, D. & Rappuoli, R. Comparison of open-source reverse vaccinology programs for bacterial vaccine antigen discovery. *Front. Immunol*. **10**, 113 (2019). 10.3389/fimmu.2019.00113 PMC6382693 30837982

[193] Gfeller, D. & Bassani-Sternberg, M. Predicting antigen presentation—what could we learn from a million peptides. *Front. Immunol.* **9**, 1716 (2018). 10.3389/fimmu.2018.01716 PMC6068240 30090105

[194] Li G, Iyer B, Prasath VBS, Ni Y, Salomonis N (2021). Deepimmuno: deep learning-empowered prediction and generation of immunogenic peptides for T-cell immunity. Brief. Bioinform..

[195] Chen B (2019). Predicting HLA class II antigen presentation through integrated deep learning. Nat. Biotechnol..

[196] Rigo MM (2015). DockTope: a web-based tool for automated pMHC-I modelling. Sci. Rep..

[197] Koşaloğlu-Yalçin Z (2022). Combined assessment of MHC binding and antigen abundance improves T cell epitope predictions. iScience.

[198] Batra R (2020). Screening of therapeutic agents for COVID-19 using machine learning and ensemble docking studies. J. Phys. Chem. Lett..

[199] Sehnal, D., Rose, A. S., Koča J., Burley, S. K. & Velankar, S. Mol*: towards a common library and tools for web molecular graphics. in *Proceedings of the Workshop on Molecular Graphics and Visual Analysis of Molecular Data*. MolVA ’18. Brno, Czech Republic. p. 29–33 (Eurographics Association, 2018).

[200] Bengio Y, Courville A, Vincent P (2013). Representation learning: a review and new perspectives. IEEE Trans. Pattern Anal. Mach. Intell..

[201] Detlefsen NS, Hauberg S, Boomsma W (2022). Learning meaningful representations of protein sequences. Nat. Commun..

[202] Kingma, D. P. & Welling, M. Auto-encoding variational Bayes. *arXiv*10.48550/arXiv.1312.6114 (2014).

[203] Goodfellow, I. J. et al. Generative adversarial nets. in *Proceedings of the 27th International Conference on Neural Information Processing Systems - Volume 2*. NIPS’14. p. 2672–2680 (MIT Press, 2014).

[204] Sohl-Dickstein, J., Weiss, E., Maheswaranathan, N. & Ganguli, S. Deep unsupervised learning using nonequilibrium thermodynamics. in *Proceedings of the 32nd International Conference on Machine Learning*. p. 2256–2265 (PMLR, 2015).

